# The Structure and Properties of Sepiolite with Partial Lattice Ions Substituted by Aluminum Ions

**DOI:** 10.3389/fchem.2021.721225

**Published:** 2021-08-25

**Authors:** Huiwen Chen, Junming Geng, Zepeng Zhang, Rui Jiang, Jingya Zhai, Jinchuan Zhang

**Affiliations:** ^1^ Beijing Key Laboratory of Materials Utilization of Nonmetallic Minerals and Solid Wastes, National Laboratory of Mineral Materials, School of Materials Science and Technology, China University of Geosciences, Beijing, China; ^2^ National Engineering Technology Research Center of Flame Retardant Materials, School of Materials, Beijing Institute of Technology, Beijing, China; ^3^ School of Energy Resources, China University of Geosciences, Beijing, China

**Keywords:** sepiolite, indigo, aluminum-modified, substitution, solid acidity

## Abstract

Sepiolite was modified with Al^3+^ via hydrothermal reaction. The substitution amount of Al^3+^ for Mg^2+^ and Si^4+^ located at sepiolite lattice and the influence of substitution amount on the structure, specific surface area, and surface acidity of Al-modified sepiolite were investigated. On this basis, indigo–sepiolite composite pigments were prepared by Al-modified sepiolite and indigo via grinding method to evaluate the influence of Al-modified sepiolite on the structure, bonding strength, and weather resistance of composite pigment. The crystal structure of Al-modified sepiolite had no obvious change after modification. Al^3+^ mainly substituted Mg^2+^ located at the octahedron of the sepiolite lattice, and the substitution amount was positively related to the dosage of Al^3+^. The specific surface area of Al-modified sepiolite decreased and the distribution of channel size became wider after Al modification. In addition, the absolute value of zeta potential decreased as well as the solid acid sites increased with the increase of Al substitution in Al-modified sepiolite. For indigo–sepiolite composite pigments, the structure of Al-modified sepiolite had no obvious change as well. The adsorption amount of indigo in composite pigment after treating by DMSO and Al content as well as weak acid amount in Al-modified sepiolite presented linear correlation, indicating that Al modification could enhance the bonding strength between indigo and Al-modified sepiolite by increasing the amount of coordinated water with Al. For indigo, Al-modified sepiolite could brighten the color and reduce the weather resistance of the prepared composite pigment. The results of this study provide a new idea and basis for regulating the structure and properties of clay and for studying the preparation of composite pigment and clay functional materials.

## Introduction

Maya Blue (MB) pigment is an organic–inorganic composite pigment that widely exists in the murals and codes of ancient Maya ruins (Gettens, 1962; Giustetto et al., 2006; Arnold et al., 2012; Doménech-Carbó et al., 2014; Buti et al., 2018). It has attracted considerable attention because of its characteristic brightness after centuries of vicissitudes (José-Yacamán et al., 1996; Vandenabeele et al., 2005; Chiari et al., 2008; Grazia et al., 2020). MB was confirmed as a complex fabricated by precipitating indigo into palygorskite. Plenty of research studies on MB mainly concerned the type of attachment between indigo and palygorskite and the location of indigo in palygorskite to reveal the mystery of its unique stability (Giustetto et al., 2005; Chiari et al., 2008).

Sepiolite is widely used to prepare composite pigment with indigo and in turn compared with MB because it has a similar structure to palygorskite (Van Olphen, 1966; Giustetto et al., 2011). The result shows that the indigo–sepiolite composite pigment is less stable than MB (Sánchez Del Río et al., 2006; Giustetto et al., 2011). Both sepiolite and palygorskite are fibrous phyllosilicate formed by two continuous [SiO_4_] tetrahedrons and an intermittent [MgO_6_] octahedron, and the periodic inversion in [SiO_4_] tetrahedron forms the channels of sepiolite and palygorskite. The difference between these two minerals is that the inverse period of [SiO_4_] tetrahedron in sepiolite is 4, while that in palygorskite is 6, resulting in larger size of channels in sepiolite. In addition, the isomorphic substitution of Al^3+^ usually occurs in [SiO_4_] tetrahedron and [MgO_6_] octahedron in the two minerals, leading to different Al contents of palygorskite and sepiolite (Giulieri et al., 2012). Therefore, the difference in stability between the indigo–sepiolite composite pigment and MB is extremely possible due to the different channel sizes and Al content of the mineral substrate.

Silicate minerals possess acid–base conjugation formed by negative charge generated by isomorphic substitution, neutralized protons, and countercations in minerals (Ramírez et al., 2011; Tazi et al., 2012). The surface acidity is essentially determined by the electronegativity of the element bonding with oxygen (Busca, 1999), and thus, the Al content will directly affect the surface acidity and basicity of sepiolite and palygorskite. The acid and base in silicate minerals show catalytic activity in organic reactions, e.g., the pyrolysis behaviors of organic matters, the methylbutynol conversion, the Biginelli type reaction of aldehydes, and the synthesis of malononitrile prepolymers (Corma and Martín-Aranda, 1993; Hu et al., 2003; Niwa et al., 2010; Pushpaletha and Lalithambika, 2011; Liu et al., 2013a; Jha et al., 2013; Novikova et al., 2013; Bu et al., 2017; Phukan et al., 2017). The acid–base conjugation in minerals plays a role to fix organic molecules during the reaction, for example, in the polymerization reaction of D, L-Lactid catalyzed by acid-modified montmorillonite, the D, L-Lactid molecules were fixed on the accessible tetrahedral Al sites (act as acid sites) through oxygen atoms (Aslya et al., 2016). The amino group in aminobenzodifurandione dye was protonated by the acid bound to the oxygen atom of silanol (Lewis base) when probing the basicity of solid acids (Spange et al., 2005). In composite materials, the combination of organic molecules and inorganic substrates is usually affected by the solid surface acid. The flavylium dye was stabilized by the solid acid that is derived from an Al impurity in silicate (Kohno et al., 2008). The azobenzene molecule was protonated by Brönsted acid in AlPO_4_-5 zeolite and then interacted with the framework oxygen atoms (Polisi et al., 2018). The acidic site montmorillonite framework played an important role to form a strong bond with thioindigo (Ramírez et al., 2011). Indigo possesses a similar structure to thioindigo, and sepiolite and palygorskite have similar acid–base conjugation to silicate minerals as well. Therefore, the acidity and basicity in sepiolite and palygorskite will also affect the bonding strength with indigo and the stability of the composite pigment, while it has not been discussed in the previous research.

The surface acidity and basicity of the minerals are affected by the modified cations since the electronegativity of cations changes. The basicity of sepiolite increased after being modified by Fe^3+^; therefore, Lewis acid CH_3_Cl had a greater affinity towards the modified sepiolite result from stronger acid–base interaction (Lazarevic et al., 2011). Two ways were used in cation modification to regulate the surface acidity and basicity of clay minerals. One is that the cations are simply adsorbed in the clay minerals. The polarization of these cations to adsorbed water molecules affects the acidity and basicity of the clay minerals. Montmorillonite, modified with Zn^2+^, Fe^3+^, and Al^3+^ through interlayer cation exchange, had increasing acidity in turn (Jha et al., 2013). Another is the substitution between free cations and lattice cations in clay minerals to change the acidity and basicity according to the electronegativity of cations. Al^3+^, Cr^3+^, H^+^, Na^+^, and La^3+^ were used to modify sepiolite, and only Al-modified sepiolite has super strength acid sites (Corma et al., 1991) since Al^3+^ was introduced in the octahedron of sepiolite, and then the catalytic activity of Al-modified sepiolite for dehydration of ethanol and gasoil cracking was improved (Corma and Perez-Pariente, 1987; Zheng et al., 2010). Therefore, the Al content in the sepiolite can be regulated by the substitution of Al for the lattice cations in sepiolite, thereby changing the surface acidity of the sepiolite and the interaction between organic and inorganic substances.

In this study, sepiolite with low content of Al was modified by hydrothermal reaction with different dosages of AlCl_3_ to regulate its solid acidity. The obtained Al-modified sepiolite was used to prepare composite pigment with indigo via the grinding method. The bonding strength between indigo and Al-modified sepiolite and the weather resistance of the composite pigment were evaluated to explore the interaction between reactants.

## Experimental

### Materials

**FIGURE 1:**
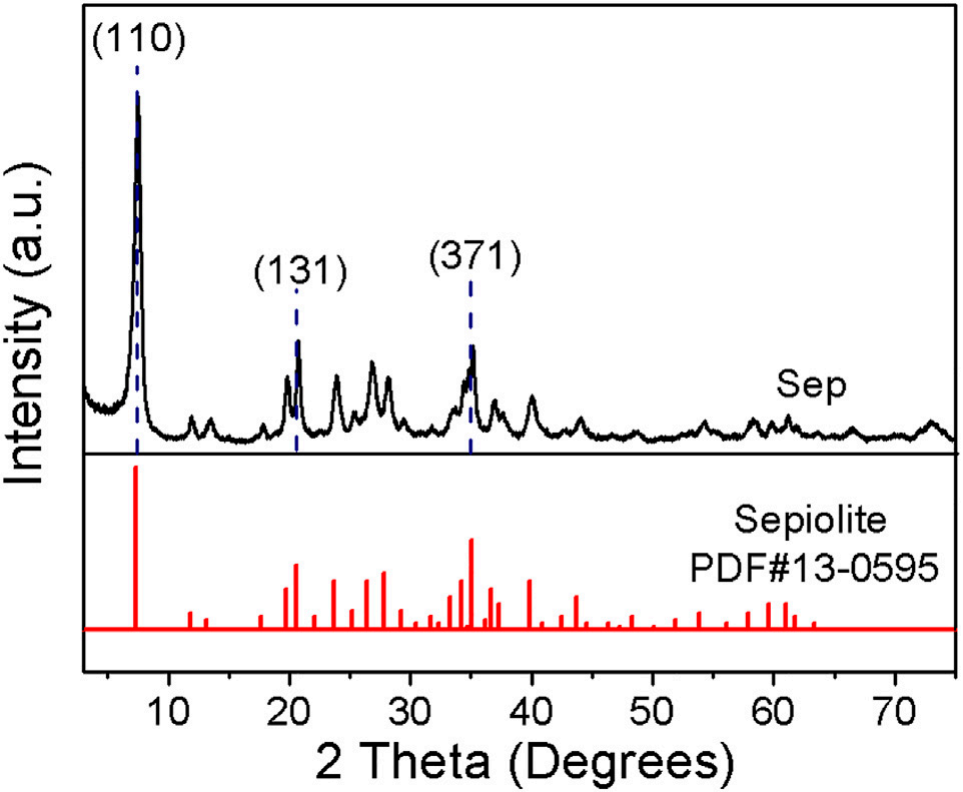
XRD pattern of Sep.

Sepiolite (Sep) was obtained from Sigma-Aldrich (Shanghai) Trading Co., Ltd. The pattern of XRD and the chemical composition are given in Figure 1 and Table 1, respectively. AlCl_3_ 6H_2_O (analytically pure) was purchased from Beijing Chemical Plant. Indigo (Id, purity of 98%) was purchased from Shanghai Macklin Biochemical Technology Co., Ltd.

**TABLE 1:** Chemical composition of Sep used in the study.

Component	Weight %
SiO_2_	69.32
MgO	25.43
Al_2_O_3_	2.89
K_2_O	0.53
Fe_2_O_3_	0.33
CaO	0.18
ZnO	0.14
TiO_2_	0.10
Others	1.08

### Preparation of Al-Modified Sep

The suspensions were prepared by mixing 7.00 g of Sep, 70 ml of the solution containing different dosages of AlCl_3_ 6H_2_O (0.00, 3.50, 7.00, and 70.0 mmol). The suspensions were kept in a 100 ml tetrafluoroethylene-lined autoclave at 120°C for 10 h. The precipitate was centrifuged and washed with deionized water until Cl^−^-ion-free and then dried at 60°C for 24 h in an oven. Finally, the dry precipitate was ground and passed through a 200-mesh sieve for further use. The resultant Al-modified Sep (Al-Sep) was marked as Al-x-Sep (x represents the amount of substance of AlCl_3_ 6H_2_O, and the unit is mmol). In addition, the supernatant after centrifugation was collected and volumed to 2 L, marked as “S”.

### Preparation of Id-Al-Sep Composite Pigments

Id was dispersed in deionized water, sonicated for 30 min in an ultrasonic cleaner (SN-4200 DTDN, Scientz, Zhejiang, China), and then shaken until the Id was evenly dispersed. 1 g of Al-x-Sep was mixed with 10 ml of Id dispersion (Id/Sep is 0.125 mmol/g) and milled in a planetary ball mill (XGB2, BYT, Jiangsu, China) at 560 rpm for 2 h. The obtained solid was dried, milled, and sieved (200-mesh sieve). The resultant composite pigment was labeled as Id-Al-x-Sep.

### Calculation of Substitution Amount of Al^3+^ for Mg^2+^ and Si^4+^ in Sep

The consumption of Al^3+^ and the amount of Mg^2+^ and Si^4+^ substituted by Al^3+^ in Sep were tested and calculated by an inductively coupled plasma emission spectroscopy (ICP, ICAP7600, Thermo, United States). 3.5 ml of AlCl_3_ solution for modification was diluted to 100 ml, marked as “Al”. The supernatant “S” and diluted AlCl_3_ solution “Al” were then diluted by the same multiple a. The obtained solution was characterized by ICP to determine the concentration of Al^3+^, Mg^2+^, and Si^4+^. The substitution amount R (mmol/g) (including the consumption of Al^3+^ and the amount of Mg^2+^ and Si^4+^ substituted by Al^3+^ in Sep) was calculated according to the following equation:

$$R=\frac{2\times \left|C_{I}^{S}-C_{I}^{Al}\right|}{M\times 7a},$$ (1)

where $C_{I}^{S}$ (ppm) and$C_{I}^{Al}$ (ppm) are the concentrations of ion I in diluted solution “S” and “Al,” respectively, M (g/mol) is the molar mass of ion I, a is the dilution multiple of solution “S” and “Al,” 7 (g) refers to the mass of Sep during the Al modification, 2 stands for the volume of solution “S” 2L, and I represents Al^3+^, Mg^2+^, and Si^4+^.

### Evaluation of Bonding Strength Between Id and Al-Sep

0.01 g of composite pigments was dispersed in 8 ml DMSO and shaken in a water bath shaker (SHA-B, Guohua, Jiangsu) at 30°C and 170 rpm for 24 h. The supernatants were separated by centrifugation and analyzed by a UV–Vis spectrophotometer (UV7600, Lengguang, Shanghai). The concentration of the supernatants was calculated by the Lambert–Beer law, and then, the adsorption amount of Id in Al-Sep was calculated to evaluate the bonding strength between Id and Al-Sep.

### Weather Resistance of Id-Al-Sep Composite Pigments

0.2 g of composite pigment was pressed into a glass groove with a diameter of 2 cm, covered by a quartz glass, and irradiated by a UV lamp (AC/90–240 V, Zigu, Guangzhou) with a wavelength of 395 nm and radiation intensity of 650 mW/cm^2^. The CIE of composite pigment irradiated by a UV lamp at different times was measured by a colorimeter (NR60CP, 3 nh, Guangzhou). The following formula was used to calculate the color difference (ΔE):

$$\Delta E=\sqrt{{\left(L_{1}-L_{0}\right)}^{2}+{\left(a_{1}-a_{0}\right)}^{2}+{\left(b_{1}-b_{0}\right)}^{2}},$$ (2)

where (L,a,b) reflects the lightness, red-green, and yellow-blue of the pigment, respectively, determining the color of the pigment. ($L_{1},a_{1},b_{1}$) and ($L_{0},a_{0},b_{0}$) are the average of three measurements collected on the pigments after and before UV irradiation, respectively. ΔE reflects the weather resistance of the composite pigments; the smaller ΔE, the better the weather resistance (Ouellet-Plamondon et al., 2015).

### Characterization

The chemical composition of Al-Sep was analyzed by an X-ray fluorescence spectrometer (XRF, XRF-1800, Shimadzu, Japan). 0.1 g of Al-Sep powder was pressed with boric acid into a tablet for analysis. The morphology of samples was characterized by a transmission electron microscopy (TEM, JEM-2100Plus, JEOL). The Fourier Transform infrared (FTIR) spectra were carried out by a FTIR spectrometer (Spectrum 100, PerkinElmer, United States) range from 1,200 to 2000 cm^−1^ with KBr background. X-ray diffraction (XRD) pattern used X-ray diffractometer (B8 Advance, Bruker, Germany) operating at Cu Kα, 40 mA, and 20 kV, scanning from 3° to 80° with a rate of 8°/min. The zeta potential (ζ) was tested by a zeta potential analyzer (Zetasizer Nano ZS90, Malvern, United Kingdom), and 0.01 g of sample was dispersed in 10 ml deionized water with sonication for 30 min. The specific surface area (BET method) of the sample was obtained from the adsorption–desorption isotherm of N_2_ measuring by a specific surface area and pore size analyzer (Autosorb IQ, Quantachrome, United States of America) at 77 K. The sample was firstly degassed at 120°C for 2 h. The pore size distribution of the sample was calculated by the density functional theory (DFT) model. The ^27^Al NMR one-pulse signal was obtained by a nuclear magnetic resonance instrument (Bruker AVANCE III 600 M, Bruker, Germany) at the frequency of 156.38 MHz and the tube diameter of 3.2 mm. The chemical shift of ^27^Al resonance line was referred to Al (NO_3_)_3_ aqueous solution. The ^27^Al experiment was recorded with a spinning rate of 15 kHz, relaxation delay of 1 s, and number of scans of 1,024. The TPD analysis was carried out by a chemical adsorption analyzer (AutoChem II 2,920, Micromeritics, United States). 0.1 g of sample was pretreated with Ar gas at 160°C, and NH_3_ was then introduced until the adsorption of the sample was saturated. Following NH_3_ adsorption, Ar gas was used to remove residual NH_3_ on the surface of the sample. Finally, the sample was degassed at a rate of 10°C/min from 50 to 600°C, and the desorbed NH_3_ at different temperatures was collected.

## Results and Discussion

### Preparation of Al-Modified Sep

#### The Influence of Al Dosage on the Composition of Al-Modified Sep

The consumption of Al^3+^ and the substitution amount of Mg^2+^ and Si^4+^ in Sep were calculated by Eq. (1), shown in Table 2. For Al-0.00-Sep, traces of Mg^2+^ and Si^4+^ exist in the solution “S,” indicating that traces of Mg^2+^ and Si^4+^ elute from the Sep during the hydrothermal reaction even without AlCl_3_. After introducing AlCl_3_, the substitution amount of Mg^2+^ and Si^4+^ (i.e., the amounts of Mg and Si dissolved from Sep) increases, demonstrating that Al^3+^ can promote the substitution of Mg^2+^ and Si^4+^ in Sep. The consumption of Al^3+^ and the substitution amount of Mg^2+^ increase, while the substitution amount of Si has no obvious change with the increase in dosage of AlCl_3_. As the dosage of AlCl_3_ reaches 70.0 mmol, the substitution amounts of Al^3+^, Mg^2+^, and Si^4+^ are 1.5831 mmol/g, 1.6756 mmol/g, and 0.1017 mmol/g, revealing that the substitution of Al^3+^ mainly occurs with Mg^2+^.

**TABLE 2:** The substitution amount (mmol/g) of Al^3+^, Mg^2+^, and Si^4+^.

Samples	Al	Mg (−)	Si (−)
Al-0.00-Sep	0.0000	0.0056	0.0197
Al-3.50-Sep	0.4785	0.6855	0.1061
Al-7.00-Sep	0.8621	1.2498	0.1709
Al-70.0-Sep	1.5831	1.6756	0.1017

(−) indicates that the ion was substituted by Al^3+^.

Table 3 shows the element content of Al-Sep modified by different dosages of AlCl_3_ characterized by XRF. As the dosage of AlCl_3_ increases, the content of Al in Sep increases, the content of Mg gradually decreases, while the content of Si has no significant change, which is consistent with the substitution amount result exhibited in Table 2. The content of Cl in Al-Sep is less than 0.10%, which is significantly lower than the content of Al in the samples, indicating that Cl^−^ adsorbed on the surface of Al-Sep during the modification process is effectively removed.

**TABLE 3:** Element content of Al-Sep modified by AlCl_3_ with different dosages.

Sample	Element content (%)							
	Si	Mg	Al	F	K	Fe	Ca	Cl
Al-0.00-Sep	32.58	15.42	1.36	0.77	0.47	0.26	0.12	0.01
Al-3.50-Sep	32.18	13.92	3.12	0.73	0.56	0.27	0.03	0.01
Al-7.00-Sep	32.30	12.37	4.51	0.67	0.43	0.25	0.02	0.01
Al-70.0-Sep	32.88	10.64	5.43	0.51	0.41	0.24	0.01	0.10

According to the calculation of the ion substitution amount and characterization of XRF, the composition of Al-Sep is mainly changed via the substitution of Al^3+^ for Mg^2+^ in Sep during the Al modification process. Since the radius of Al^3+^ is smaller than that of Mg^2+^ and the charge of Al ^3+^ is larger than that of Mg^2+^, the substitution of Al^3+^ for Mg^2+^ can reduce the energy and stabilize the system (Liao and Xia, 2013).

#### The Structure of Al-Modified Sep

**FIGURE 2:**
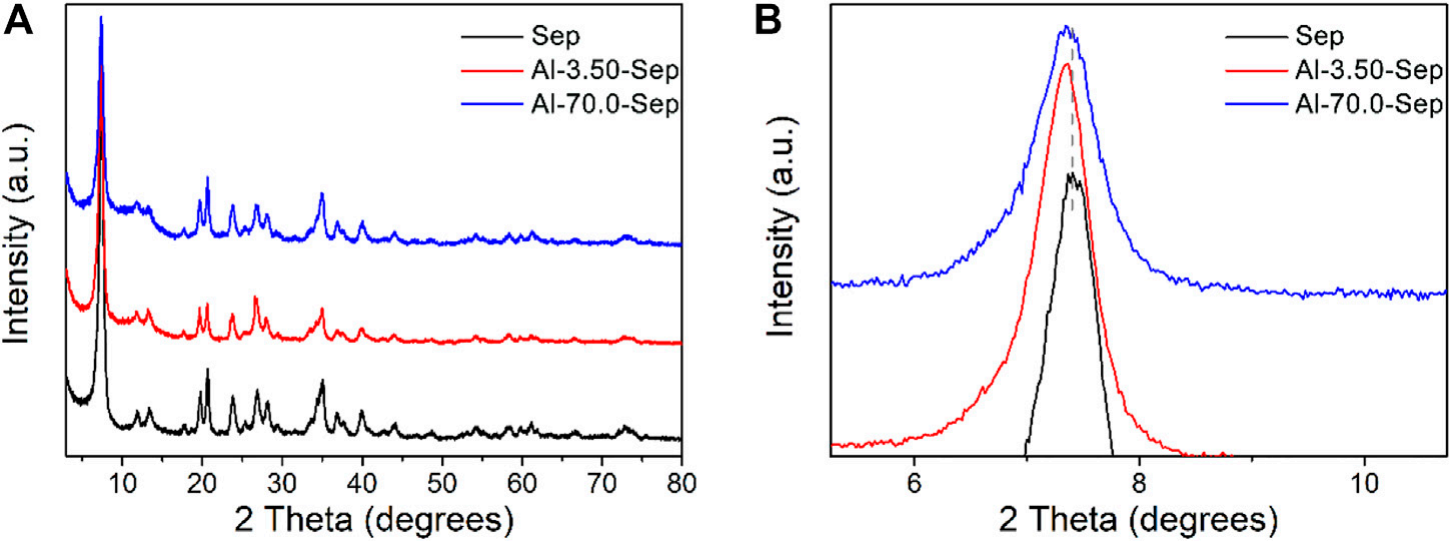
**(A)** XRD patterns of Sep, Al-3.50-Sep, and Al-70.0-Sep. **(B)** Partially enlarged view of Figure 2A.

Figure 2 presents the XRD patterns of Sep and Al-Sep. The typical reflection peaks of Sep are located at 2θ = 7.45°, 20.53°, and 34.92°corresponding to (110), (131), and (371) planes, according to JCPDS card no. 13-0595 (Figure 1), without other impurity peaks. The peaks of Al-Sep are identical with that of Sep (Figure 2A), suggesting that the fiber structure of sepiolite has no significant change, and no impurity crystal is formed during the modification. The reflection peaks at a small angle of Al-Sep shift to a low angle slightly compared with that of Sep, and as the dosage of AlCl_3_ increases, the shift angle increases (Figure 2B). The diffraction angles at (110) plane in Sep, Al-3.50-Sep, and Al-70.0-Sep are 7.45°, 7.43°, and 7.34°, respectively, corresponding to the interplanar spacing of 11.85 Å, 11.89 Å, and 12.03 Å, revealing that the interplanar space of (110) plane increases as the dosage of Al increases. The change in the interplanar space and the difference between the radii of Al^3+^ and Mg^2+^are in the same order of magnitude; therefore, the shift of diffraction angle at (110) plane is attributed to a slight distortion of the sepiolite during the modification process.

#### The Morphology of Al-Modified Sep

**FIGURE 3:**
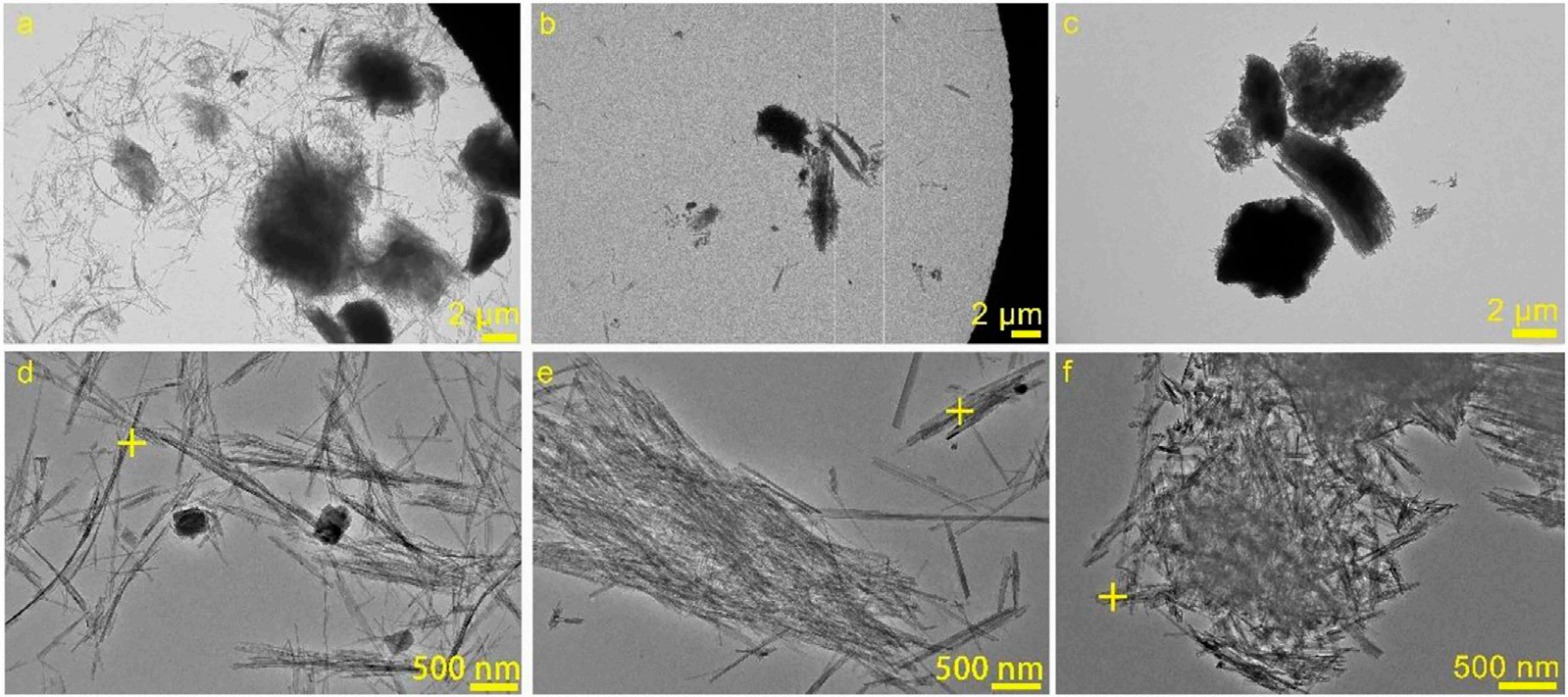
TEM images of **(A, D)** Sep, **(B, E)** Al-3.50-Sep, and **(C, F)** Al-70.0-Sep.

Figure 3 provides the TEM images of Sep, Al-3.50-Sep, and Al-70.0-Sep. The length of Sep is about 0.5–6 μm, the width is about 20 nm, and Sep mainly exists as single nanofibers (Figure 3A, D). As illustrated in Figure 3B, C, the fibrous structure of sepiolite has not been destroyed after being modified by AlCl_3_ while the single fibers obviously decrease, indicating that Al modification will reduce the dispersibility of sepiolite. From the results of EDS-point (Table 4) on the sepiolite fibers marked in Figure 3D–F, the main elements on the fiber in Sep are O, Si, and Mg, and the elements Al and Cl appear on the fiber of Al-3.50-Sep after introducing AlCl_3_. The content of Al in Al-70.0-Sep is significantly increased, demonstrating that the element Al exists on the fibers of sepiolite during the hydrothermal reaction with AlCl_3_, and the content increases with the increase of the dosage of AlCl_3_.

**TABLE 4:** The corresponding EDS results marked in Figures 3D–F.

Samples	Al	Mg	Si	O	Cl
Sep	0	10.30	27.09	44.41	0
Al-3.50-Sep	1.78	9.70	33.37	55.10	0.05
Al-70.0-Sep	3.12	6.97	33.72	56.18	0.00

#### The Substitution Position of Al^3+^

**FIGURE 4:**
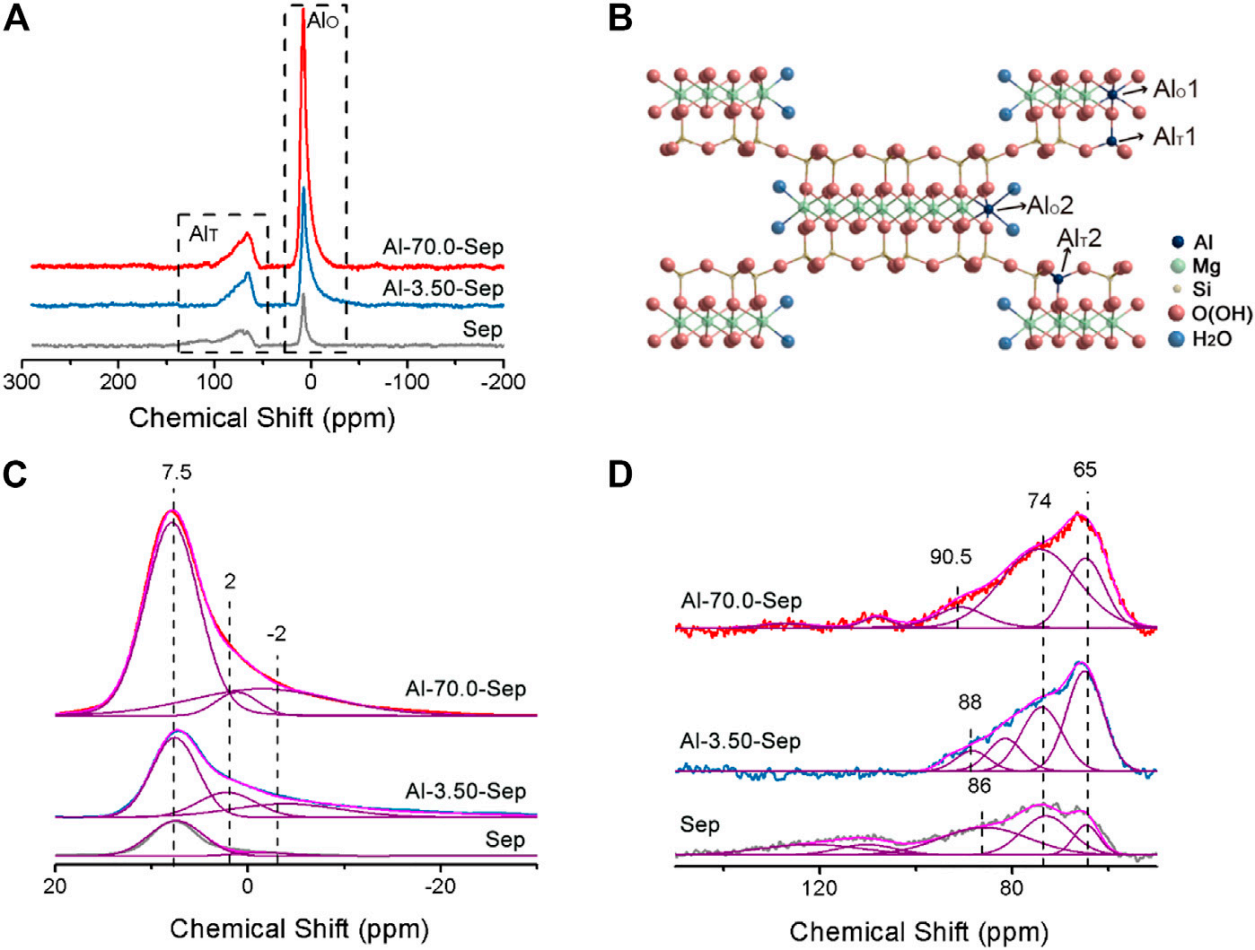
**(A)**^27^ Al NMR spectra of Sep, Al-3.50-Sep, and Al-70.0-Sep. **(B)** Schematic diagram of the position of Al_T_ and Al_O_ in sepiolite. The simulated results of (**A**) **(C)** at 20∼-30 ppm and **(D)** at 150∼50 ppm.

The substitution position of Al^3+^ was distinguished by solid-state ^27^Al NMR spectra, shown in Figure 4. The signals are observed at ∼7 and ∼70 ppm, attributed to octahedral [AlO_6_] (Al_T_) and tetrahedral [AlO_4_] (Al_O_) in the structure of sepiolite, respectively (Caillerie and Fripiat, 1992; Komarneni et al., 1986). The Al_T_ and Al_O_ in Sep occur due to amorphous impurity and the substitution of Al^3+^ for Mg^2+^ located at [MgO_6_] and Si^4+^ located at [SiO_4_] in sepiolite lattice. The intensity of ^27^Al NMR signal reflects the content of Al, and the peak area of the signal can be integrated to calculate the content of Al at different positions, shown in Figure 4C, D. The width of the signal reflects the symmetry of Al complex, and the narrower spectrum reflexes the higher symmetry of the complex (Wang et al., 2000).

For Sep, two bands of Al_O_ at 7.5 ± 0.5 ppm and −2 ± 1 ppm, deriving from the Al at the edge (Al_O_2) and the section (Al_O_1) of the octahedron in sepiolite, and two signals of Al_T_ at 65 ± 1 ppm and 74 ± 1 ppm, corresponding to the Al at the center (Al_T_2) and edge (Al_T_1) of tetrahedron in sepiolite, are very close to the previous literature, marked in Figure 4B (Caillerie and Fripiat, 1992; Wang et al., 2000; Aslya et al., 2016). The signals at 86 ± 5 ppm are broad, revealing poor symmetry, which might be from the Al in the tetrahedron in the amorphous impurity (Wang et al., 2000). The ^27^Al signals in Sep come from the impurity and the substitution of Mg^2+^ in the octahedron and Si^4+^ in tetrahedron by Al^3+^ during the mineralization process (Zhuang et al., 2019).

The intensity of ^27^Al signals of Al-3.50-Sep and Al-70.0-Sep is higher than that of Sep, illustrating the content of Al increases after modification, which is consistent with the result of ICP and XRF. Al-3.50-Sep and Al-70.0-Sep have similar chemical shifts to Sep, revealing that Al^3+^ can enter the tetrahedron and octahedron in sepiolite, rather than just forming oxides or adsorbing on the surface of sepiolite. Al-Sep has a signal at 2 ± 1 ppm that is absent in Sep, which belongs to the chemical shift for amorphous aluminum silicate formed during the hydrothermal reaction. The signal of Al-0.05-Sep at 86 ± 5 ppm is divided into two signals, which are attributed to the change of the impurity in sepiolite and then the formation of two kinds of Al_T_ with different chemical environments.

The content of Al at different positions in sepiolite of samples is calculated and shown in Table 5. The Al content at −2 ± 1 ppm and 7.5 ± 0.5 ppm increased significantly as the dosage of AlCl_3_ increases, indicating that Al^3+^ mainly substitutes the Mg^2+^ at the edge and section of the octahedron in sepiolite. Al^3+^ can substitute Si^4+^ in tetrahedron as well, but the substitution amount is obviously lower than that occurs in the octahedron. In addition, the signals at 108 and 127 ppm are the chemical shift produced by the tetrahedral coordination formed by Al^3+^, Cl^−^, and F^−^ adsorbed on the surface of sepiolite (Wang et al., 2000).

**TABLE 5:** The calculated results of ^27^Al NMR spectra of Sep, Al-3.50-Sep, and Al-70.0-Sep.

Samples	Al (wt.%)	Al_T_ (wt.%)	Al_T_ (ppm)			Al_O_ (wt.%)	Al_O_ (ppm)		
			65	74	86		−2	2	7.5
Sep	1.53	0.69	0.10	0.26	0.33	0.57	0.05	0	0.52
Al-3.50-Sep	3.12	1.09	0.49	0.35	0.26	1.77	0.42	0.38	0.97
Al-70.0-Sep	5.43	1.40	0.48	0.76	0.15	3.91	1.03	0.20	2.69

#### The Specific Surface Area and Micropores of Al-Modified Sep

**FIGURE 5:**
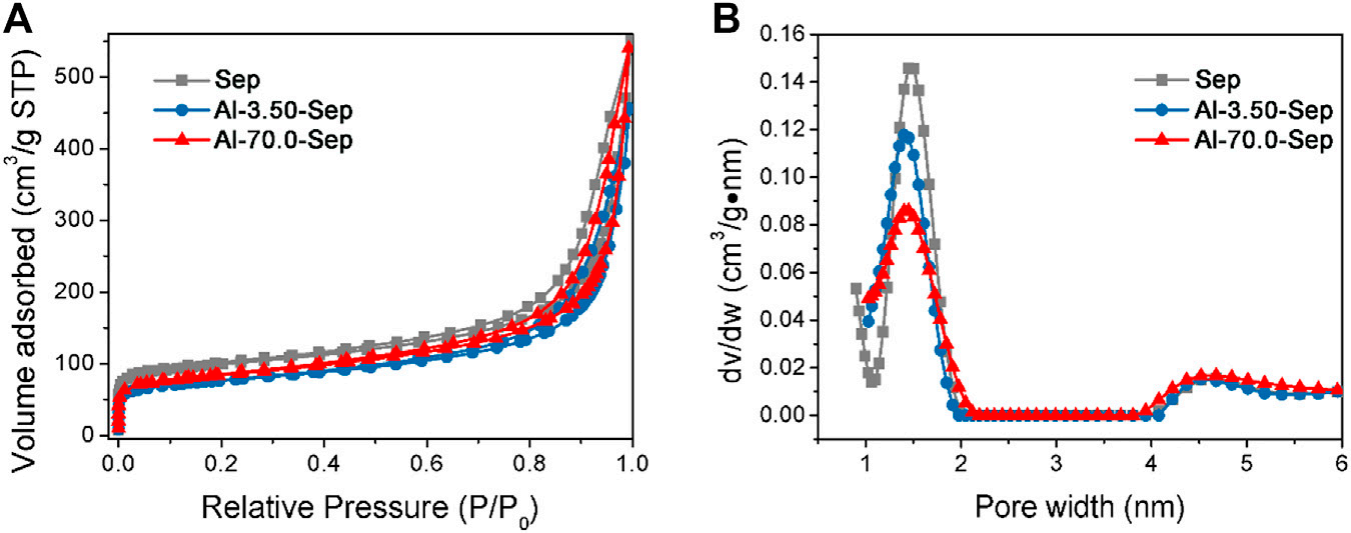
**(A)** N_2_ adsorption–desorption isotherms and **(B)** the pore size distribution plots of Sep, Al-3.50-Sep, and Al-70.0-Sep.

**FIGURE 6:**
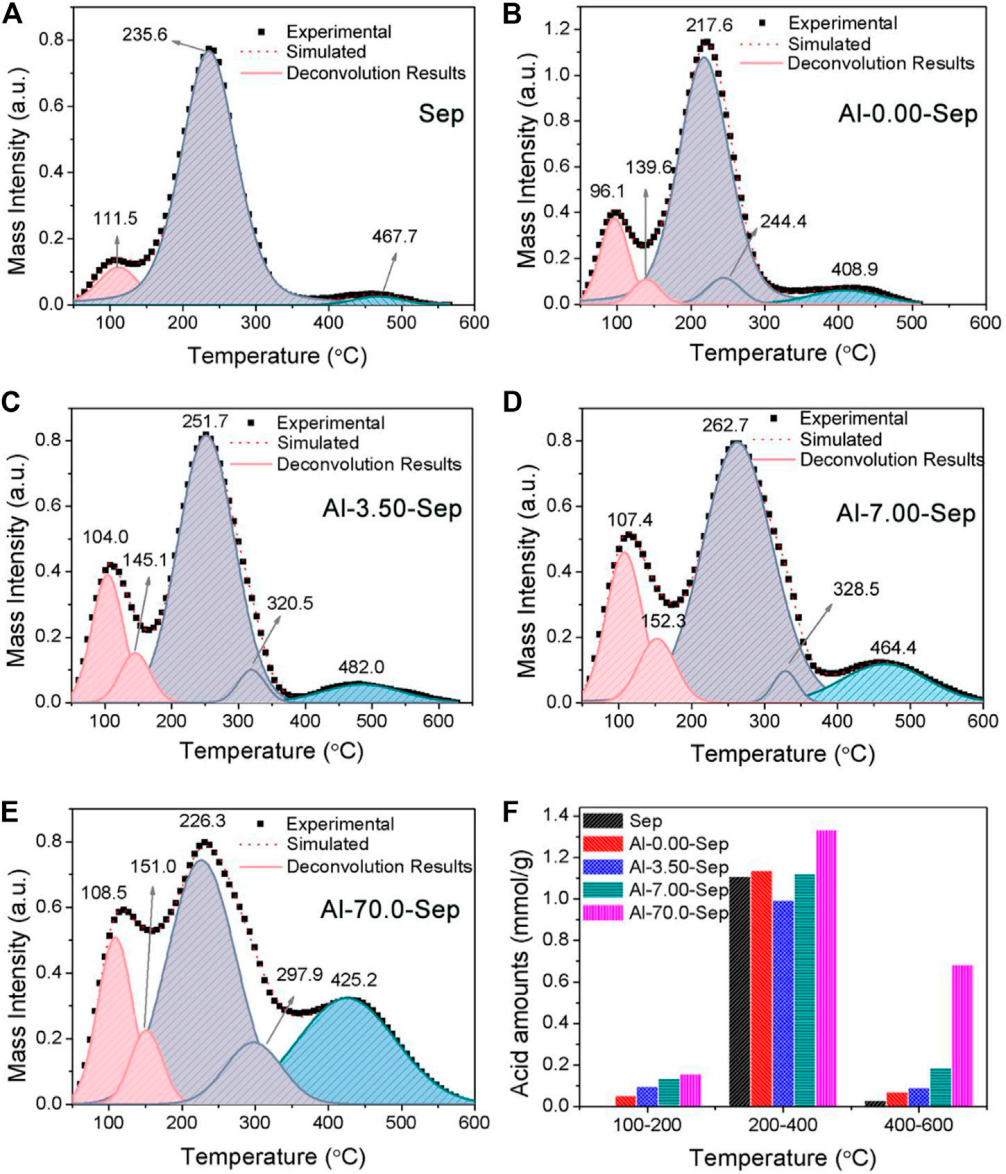
The NH_3_-TPD curves after heating from 50 to 600°C of **(A)** Sep, **(B)** Al-0.00-Sep, **(C)** Al-3.50-Sep, **(D)** Al-7.00-Sep, and **(E)** Al-70.0-Sep. **(F)** The acid amounts of samples.

The N_2_ adsorption-desorption isotherms and the pore size distribution plots of Sep, Al-3.50-Sep, and Al-70.0-Sep are shown in Figure 5. As illustrated in Figure 6A, the N_2_ amounts adsorbed on Al-3.50-Sep and Al-70.0-Sep are smaller than that adsorbed on Sep, indicating they have a smaller specific surface area, as the dispersibility of sepiolite rapidly reduces after Al modification (exhibited in Figure 3). The micropores ranging from 1 to 2 nm represent the channels of sepiolite formed by the periodic reversal of [SiO_4_] (Chen et al., 2019). As the dosage of AlCl_3_ increases, the distribution of channel size becomes wide, since the substitution of Al^3+^ for Mg^2+^ at the edge and section of the octahedron, i.e., the edge of sepiolite channel, makes the distribution of sepiolite channel size becoming uneven.

#### The Zeta Potential of Al-Modified Sep

Table 6 gives the zeta potential of Al-Sep. The following possibilities will occur during the Al modification: 1) the substitution of Al^3+^ for Mg^2+^ in the octahedron will reduce the negative surface charges; 2) the substitution of Al^3+^ for Si^4+^ in tetrahedron will increase the negative surface charges; 3) the formation of vacancy defects in the octahedron and tetrahedron will increase the negative surface charges; and 4) the adsorption of Al^3+^ on sepiolite surface will reduce the negative charges. These actions conduct at the same time and affect the final zeta potential of the sepiolite. The result shows that the absolute value of the zeta potential decreases with the increase of dosage of AlCl_3_, since the substitution of Al^3+^ mainly occurs on the octahedron in sepiolite and reduces the negative charge on the sepiolite surface.

**TABLE 6:** The zeta potential of Al-Sep modified with different dosages of AlCl_3_.

Samples	Al-0.00-sep	Al-3.50-sep	Al-7.00-sep	Al-70.0-sep
ζ (mV)	−13.5	−10.8	−9.25	−6.17

#### The Solid Acidity of Al-Modified Sep

NH_3_-TPD has been verified to characterize the solid acidity of clay mineral quantitatively by calculating relative areas of resolved bands. The acid strength is positively correlated with the desorption temperature of NH_3_ (Liu et al., 2013b). The acidity of montmorillonite is formed by the water polarized by cations, Si-OH and H_3_O^+^ adsorbed on the negative charges, unsaturated Al^3+^ in the octahedron, and adsorbed water on unsaturated Al^3+^ (marked as Al-OH_2_) (Heller-Kallai, 2006; Liu et al., 2013b; Liu et al., 2011). Si-OH, H_3_O^+^, and Al-OH_2_ behave as weak-strength acid sites (Hair and Hertl, 1970). The polarized water and unsaturated Al^3+^ behave as medium-strength acid site. The water polarized by adsorbed cations, e.g., Al^3+^, Ca^2+^, and Fe^3+^, will be transformed into exposed hydroxy and then behaves as strong-strength acid site after heating at 160°C (Liu et al., 2013b; Kaufhold et al., 2011). The acid sites of sepiolite are similar to montmorillonite; therefore, the bands of NH_3_-TPD curves of samples (shown in Figure 6A–E) at 100–200°C, 200–400°C, and 400–600°C correspond to weak-strength, medium-strength, and strong-strength acid sites, respectively. In addition, the desorption temperature occurs at ∼100°C attributed to physical adsorbed and hydrogen-bound NH_3_ (Liu et al., 2011; Liu et al., 2013b). The total acid amounts of Sep, Al-0.00-Sep, Al-3.50-Sep, Al-7.00-Sep, and Al-70.0-Sep are 1.137, 1.273, 1.177, 1.440, and 2.220 mmol/g. The amounts of weak-strength and strong-strength acid sites increase while the amount of medium-strength acid sites firstly decreases and then increases with the increase of the dosage of AlCl_3_ (Figure 6F).

Compared with Sep, the acid amount in Al-0.00-Sep increases, since Mg^2+^ and Si^4+^ in sepiolite lattice are dissolved out during the hydrothermal reaction, resulting in the increase of negative charge and the adsorbed H_3_O^+^. The grinding process during the preparation of Al-0.00-Sep can also increase acid sites by increasing the unsaturated cations and then enhancing the polarization of adsorbed water.

The weak-strength and strong-strength acid sites increase while the medium-strength acid sites reduce in Al-3.50-Sep, since the Al-OH_2_ at the edge of the octahedron and exposed octahedral Al-OH increase and the unsaturated Al^3+^ decreases (Figure 4C) after being modified by 3.50 mmol AlCl_3_. With the increase in dosage, the Al-OH_2_, unsaturated Al^3+^, and exposed Al-OH increase, and thus, the weak-, medium-, and strong-strength acid amounts are improved. TPD results illustrate that Al modification will increase the acid sites of sepiolite.

### The Id-Al-Sep Composite Pigments

#### The Structure of Id-Al-Sep Composite Pigments

##### X-Ray Diffraction

**FIGURE 7:**
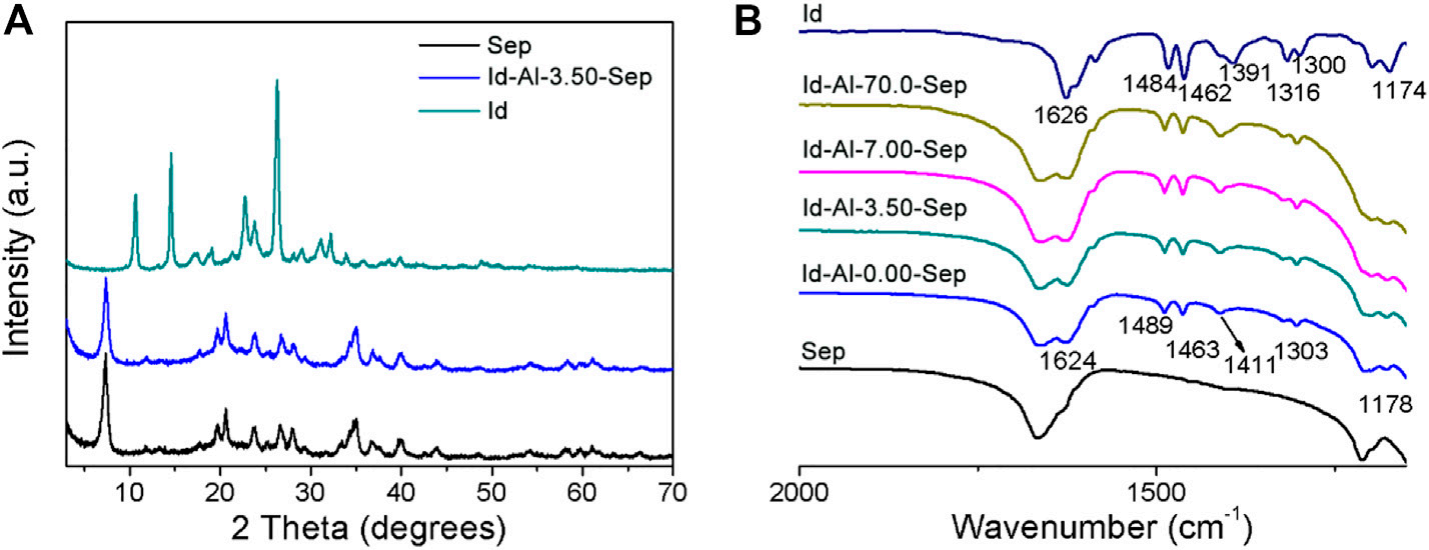
**(A)** XRD pattern and **(B)** FTIR spectra of Sep, Id, and composite pigment prepared by Id and Al-Sep.

Id was employed as the color giving agent and ground with sepiolite modified with different dosages of AlCl_3_ to prepare composite pigments. The structure of composite pigments was characterized by XRD, shown in Figure 7A. The typical reflection peaks of Id-Al-3.50-Sep are identical with that of Sep, and no obvious reflection belonging to Id is observed in Id-Al-3.50-Sep, revealing that the Id molecule is uniformly distributed on Al-Sep after grinding.

##### Fourier Transform Infrared

Figure 7B shows the FTIR spectra of Id, Sep, and composite pigments, and the stretching vibration of C=O and N-H at 1,626 cm^−1^ redshifts to 1,624 cm^−1^, corresponding to the shift caused by the formation of hydrogen bond between indigo and two water molecules which were calculated by the literature (Giustetto et al., 2005), demonstrating that C=O and N-H in Id can bond with the coordinated water, silanol, and zeolite water in Al-Sep through hydrogen bond. The vanishment of the vibration of 1,391 cm^−1^ attributed to crystalline indigo indicates the absence of crystalline indigo in composite pigments (Giulieri et al., 2012).

As illustrated by the results of XRD and FTIR, the presence of crystalline indigo is not detected in the composite pigment, indicating Id molecules reorganize on the Al-Sep during ball milling. Id molecules bond through hydrogen bond with coordinated water, silanol, and zeolite water in Al-Sep.

##### The Bonding Strength Between Id and Al-Sep

**FIGURE 8:**
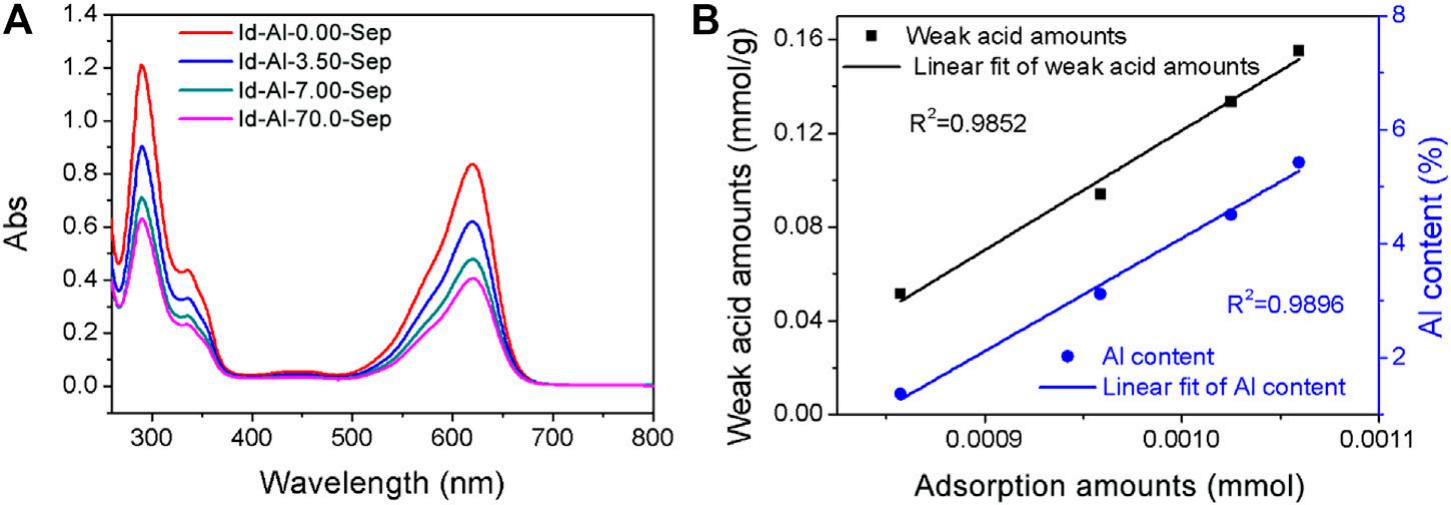
**(A)** The UV-Vis spectra of supernatant of composite pigments desorbed in DMSO. **(B)** Linear correlation between the amount of weak acid, Al content in Al-Sep, and the adsorption amount of Id in composite pigment desorbed by DMSO.

The composite pigments were desorbed by DMSO to evaluate the effect of Al modification on bonding strength between Id and Al-Sep. The removal of Id by DMSO can be an indication of available Id in Al-Sep with different bonding strengths, and the UV–Vis spectra of supernatant after desorption are shown in Figure 8A. As the dosage of modified AlCl_3_ increases, the Al content in sepiolite increases, and the desorption number of composite pigments in DMSO decreases, indicating that the bonding strength between Id and Al-Sep increases. Compared with the results of specific surface area, the possibility that the channels in Al-Sep bound the Id molecule to enhance the bonding has been eliminated. By comparison, a linear correlation is found between the Al content of Al-Sep and the adsorption amount of Id in composite pigment desorbed by DMSO. Furthermore, weak acid amount and adsorption amount of Id displayed a similar correlation (Figure 8B). The general trend is similar to the result of previous studies (Ramírez et al., 2011).

**FIGURE 9:**
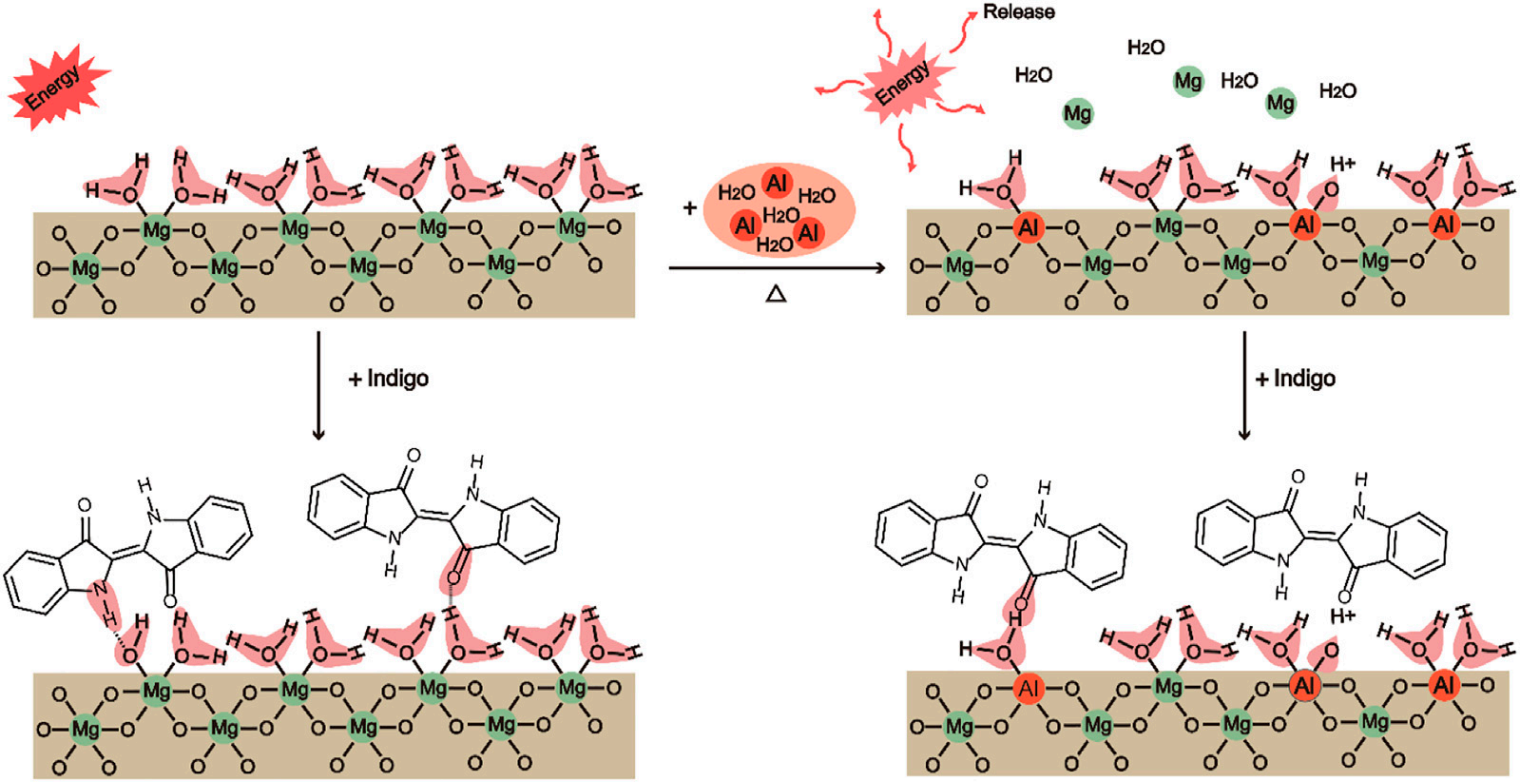
The mechanism of the substitution of Al^3+^ for Mg^2+^ located at the octahedron in sepiolite and the bonding of Sep, Al-Sep with Id (executed in (200) plane).

Weak acid sites consist of Si-OH, H_3_O^+^, and Al-OH_2_. Al modification can enhance the bonding strength between Id and Al-Sep, as the substitution of Al^3+^ mainly occurs with Mg^2+^ in the octahedron, causing the increase of Al-OH_2_ at the edge of the octahedron, and Al^3+^ has a higher electronegativity than Mg^2+^ and has a higher polarization of coordinated water, resulting in a stronger combination between C=O and N-H in Id and Al-OH_2_ in Al-Sep, and the mechanism is shown in Figure 9.

Combining the results of FTIR and adsorption amount of Id in composite pigment treated by DMSO, Id bonds with sepiolite through hydrogen bonds between C=O and N-H in Id and Si-OH, H_3_O^+^, Mg-OH_2_, and Al-OH_2_ in Al-Sep, which is consistent with the result of the previous literature (Zhuang et al., 2019). Among them, the bond energy formed by Al-OH_2_ and Id is higher than that of Mg-OH_2_. Al modification can increase the bonding strength of Id and Al-Sep by the substitution of Al^3+^ for Mg^2+^ in the octahedron.

#### The Chromaticity and Weather Resistance of Composite Pigments

##### Chromaticity

**FIGURE 10:**
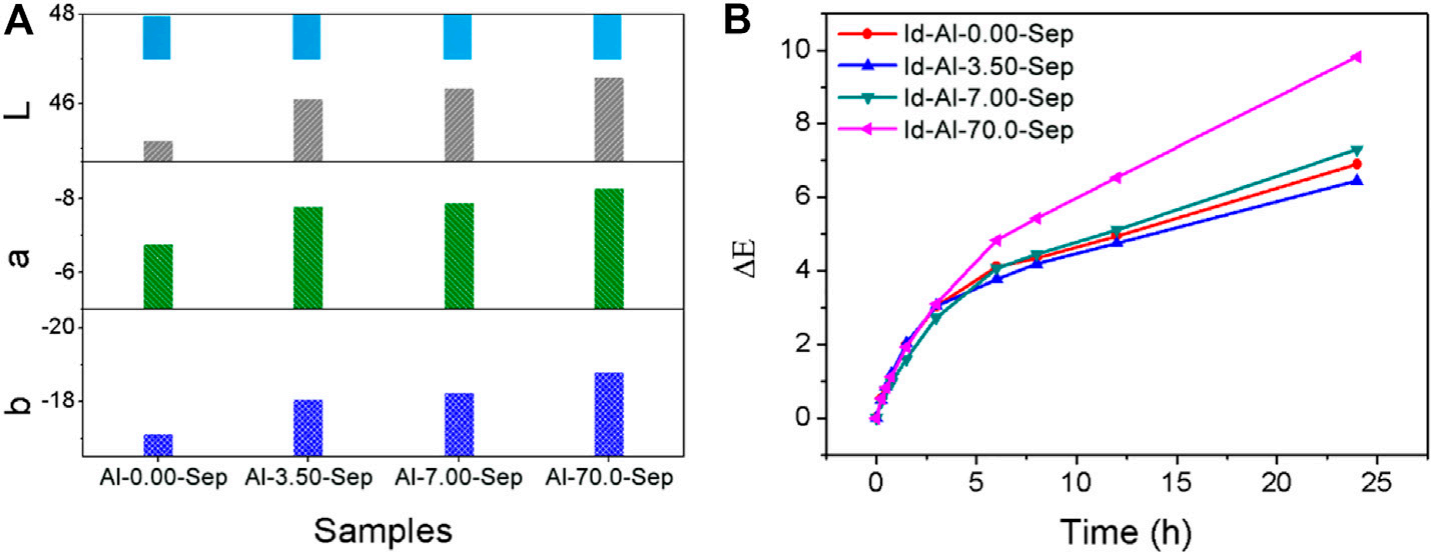
**(A)** The chromaticity of the composite pigments. **(B)** The ΔE curves of composite pigments at different irradiation times.

The chromaticity of Id-Al-Sep composite pigments is presented in Figure 10A. The absolute value of L, a, and b increases as the dosage of AlCl_3_ rises, indicating a more vivid color of composite pigments as the bonding strength between Id and sepiolite increases. Increasing the bonding strength between Id and sepiolite is an effective way to improve the chromaticity of composite pigments.

##### Weather Resistance

ΔE results of composite pigments were measured and calculated after UV irradiation at different times during 24 h (Figure 10B). A smaller ΔE performs better weather resistance. Obviously, the weather resistance of composite pigments is as follows: Id-Al-3.50-Sep > Id-Al-0.00-Sep > Id-7.00-Sep > Id-70.0-Sep shows a positive correlation with the acid amounts in Al-Sep. Intramolecular proton transfer between C=O and N-H in the Id molecule is the main pathway for Id to decolor under UV irradiation (Rondão et al., 2010). The acid in Al-Sep will promote this proton transfer in Id under UV irradiation and promotes the fading rate of Id and reduces the weather resistance of composite pigments.

In summary, Al modification can increase the amount of weak acid Al-OH_2_ in sepiolite. The Al-OH_2_ enhances the combination of sepiolite and indigo. For indigo, the acid in Al-Sep can brighten the color and accelerate the fading of composite pigments. This phenomenon originates from the special structure of indigo, and there may be different results in composite pigments prepared by other dyes.

## Conclusion

In this study, AlCl_3_ was used to modify sepiolite. The substitution of Al^3+^ mainly occurs with Mg^2+^ located at the edge and section of the octahedron in sepiolite lattice and the substitution amount increases with the increase in the dosage of AlCl_3_, since the substitution can reduce the energy of the system. The absolute value of zeta potential and specific surface area of sepiolite decreases, while the solid acidity of sepiolite increases as the dosage of AlCl_3_ rises. Al modification can enhance the bonding between indigo and sepiolite by increasing the amount of weak acid Al-OH_2_ through the substitution of Al^3+^ for Mg^2+^ located at the octahedron of sepiolite lattice. The electronegativity of Al^3+^ is greater than that of Mg^2+^, and thus, indigo can form a stronger bonding with Al-OH_2_ than that with Mg-OH_2_. Enhancing the bonding strength between indigo and sepiolite can brighten the composite pigment. The acid in sepiolite will provide a pathway to decolor by promoting the intramolecular proton transfer in the Id molecule, resulting in worse weather resistance. This conclusion is for indigo, and the protective effect of Al modification on other dyes needs further discussion.

The solid acidity and basicity of sepiolite can be regulated by changing the content and position of Al, and the catalytic activity and adsorption performance of sepiolite will be adjusted, accordingly widening the application of sepiolite in the fields of catalysis and adsorption.

## Data Availability

The original contributions presented in the study are included in the article/supplementary material; further inquiries can be directed to the corresponding author.

## References

[B1] Arnold D. E. Bohor B. F. Neff H. Feinman G. M. Williams P. R. Dussubieux L. (2012). The First Direct Evidence of Pre-columbian Sources of Palygorskite for Maya Blue. J. Archaeological Sci. 39 (7), 2252–2260. 10.1016/j.jas.2012.02.036

[B2] Aslya E. Harrane A. Belbachir M. (2016). Polymerization of DL-Lactide Induced by Protonated Montmorillonite clay as a Solid Catalyst: Mechanism Study. Mat. Res. 19 (1), 132–137. 10.1590/1980-5373-mr-2015-0322

[B3] Bu H. Yuan P. Liu H. Liu D. Liu J. He H. (2017). Effects of Complexation between Organic Matter (OM) and clay mineral on OM Pyrolysis. Geochimica et Cosmochimica Acta 212, 1–15. 10.1016/j.gca.2017.04.045

[B4] Busca G. (1999). The Surface Acidity of Solid Oxides and its Characterization by IR Spectroscopic Methods. An Attempt at Systematization. Phys. Chem. Chem. Phys. 1 (5), 723–736. 10.1039/a808366e

[B5] Buti D. Domenici D. Grazia C. Ostapkowicz J. Watts S. Romani A. (2018). Further Insight into Mesoamerican Paint Technology: Unveiling the Colour Palette of the Pre-columbian Codex Fejérváry-Mayer by Means of Non-invasive Analysis. Archaeometry 60 (4), 797–814. 10.1111/arcm.12341

[B6] Caillerie J. B. E. Fripiat J. J. (1992). Al Modified Sepiolite as Catalyst or Catalyst Support. Catal. Today 14 (2), 125–140.

[B7] Chen H. Zhang Z. Zhuang G. Jiang R. (2019). A New Method to Prepare 'Maya Red' Pigment from Sepiolite and Basic Red 46. Appl. Clay Sci. 174, 38–46. 10.1016/j.clay.2019.03.023

[B8] Chiari G. Giustetto R. Druzik J. Doehne E. Ricchiardi G. (2008). Pre-columbian Nanotechnology: Reconciling the Mysteries of the Maya Blue Pigment. Appl. Phys. A 90 (1), 3–7. 10.1007/s00339-007-4287-z

[B9] Corma A. Fornes V. Mifsud A. (1991). Aluminum-Exchanged Sepiolite as a Component of Fluid Cracking Catalysts. Washington, DC: American Chemical Society.

[B10] Corma A. Martín-Aranda R. M. (1993). Application of Solid Base Catalysts in the Preparation of Prepolymers by Condensation of Ketones and Malononitrile. Appl. Catal. A: Gen. 105 (2), 271–279. 10.1016/0926-860x(93)80252-l

[B11] Corma A. Perez-Pariente J. (1987). Catalytic Activity of Modified Silicates: I. Dehydration of Ethanol Catalysed by Acidic Sepiolite. Clay miner. 22 (4), 423–433. 10.1180/claymin.1987.022.4.06

[B12] Doménech-Carbó A. Doménech-Carbó M. T. Vidal-Lorenzo C. Vázquez de Agredos-Pascual M. L. Osete-Cortina L. Valle-Algarra F. M. (2014). Discovery of Indigoid-Containing clay Pellets from La Blanca: Significance with Regard to the Preparation and Use of Maya Blue. J. archaeological Sci. 41, 147–155. 10.1016/j.jas.2013.08.007

[B13] Gettens R. J. (1962). Maya Blue: an Unsolved Problem in Ancient Pigments. Am. Antiquity 2 (1), 557–564. 10.2307/277679

[B14] Giulieri F. Ovarlez S. Chaze A. M. (2012). Indigo/sepiolite Nanohybrids: Stability of Natural Pigments Inspired by Maya Blue. Int. J. nanotechnology 9 (3-7), 605–617. 10.1504/IJNT.2012.045334

[B15] Giustetto R. Wahyudi O. Corazzari I. (2011). Chemical Stability and Dehydration Behavior of a Sepiolite/indigo Maya Blue Pigment. Appl. Clay Sci. 52 (1-2), 41–50. 10.1016/j.clay.2011.01.027

[B16] Giustetto R. Levy D. Chiari G. (2006). Crystal Structure Refinement of Maya Blue Pigment Prepared with Deuterated Indigo, Using Neutron Powder Diffraction. European. J. Minerol. 18 (5), 629–640. 10.1127/0935-1221/2006/0018-0629

[B17] Giustetto R. Llabrés i Xamena F. X. Ricchiardi G. Bordiga S. Damin A. Gobetto R. (2005). Maya Blue: A Computational and Spectroscopic Study. J. Phys. Chem. B 109 (41), 19360–19368. 10.1021/jp048587h 16853500

[B18] Grazia C. Buti D. Amat A. Rosi F. Romani A. Domenici D. (2020). Shades of Blue: Non-invasive Spectroscopic Investigations of Maya Blue Pigments. From Laboratory Mock-Ups to Mesoamerican Codices[J]. Heritage Sci. 8 (1), 1–20. 10.1186/s40494-019-0345-z

[B19] Hair M. L. Hertl W. (1970). Acidity of Surface Hydroxyl Groups. J. Phys. Chem. 74 (1), 91–94. 10.1021/j100696a016

[B20] Heller-Kallai L. (2006). Chapter 7.2 Thermally Modified Clay Minerals. Dev. clay Sci. 1, 289–308. 10.1016/s1572-4352(05)01009-3

[B21] Hu C. Yu J. C. Hao Z. Wong P. K. (2003). Effects of Acidity and Inorganic Ions on the Photocatalytic Degradation of Different Azo Dyes. Appl. Catal. B: Environ. 46 (1), 35–47. 10.1016/s0926-3373(03)00139-5

[B22] Jha A. Garade A. C. Shirai M. Rode C. V. (2013). Metal Cation-Exchanged Montmorillonite clay as Catalysts for Hydroxyalkylation Reaction. Appl. clay Sci. 74, 141–146. 10.1016/j.clay.2012.10.005

[B23] José-Yacamán M. Rendón L. Arenas J. Serra Puche M. C. (1996). Maya Blue Paint: An Ancient Nanostructured Material. Science 273 (5272), 223–225. 10.1126/science.273.5272.223 8662502

[B24] Kaufhold S. Stanjek H. Penner D. Dohrmann R. (2011). The Acidity of Surface Groups of Dioctahedral Smectites. Clay miner. 46 (4), 583–592. 10.1180/claymin.2011.046.4.583

[B25] Kohno Y. Tsubota S. Shibata Y. (2008). Enhancement of the Photostability of Flavylium Dye Adsorbed on Mesoporous Silicate J. Microporous Mesoporous Materials 116 (1-3), 70–76. 10.1016/j.micromeso.2008.03.014

[B26] Komarneni S. Fyfe C. A. Kennedy G. J. (1986). Detection of Nonequivalent Si Sites in Sepiolite and Palygorskite by Solid-State 29Si Magic Angle Spinning-Nuclear Magnetic Resonance. Clays and Clay Minerals 34 (1), 99–102. 10.1346/ccmn.1986.0340113

[B27] Lazarevic S. Jankovic-Castvan I. Onjia A. (2011). Surface Characterization of Iron-Modified Sepiolite by Inverse Gas Chromatography. Ind. Eng. Chem. Res. 50 (20), 11467–11475. 10.1021/ie200595n

[B28] Liao L. Xia Z. (2013). Crystal Chemistry and Crystal Physics. Beijing, China: Science Press.

[B29] Liu D. Yuan P. Liu H. Cai J. Qin Z. Tan D. (2011). Influence of Heating on the Solid Acidity of Montmorillonite: A Combined Study by DRIFT and Hammett Indicators. Appl. Clay Sci. 52 (4), 358–363. 10.1016/j.clay.2011.03.016

[B30] Liu D. Yuan P. Liu H. Cai J. Tan D. He H. (2013). Quantitative Characterization of the Solid Acidity of Montmorillonite Using Combined FTIR and TPD Based on the NH3 Adsorption System. Appl. Clay Sci. 80-81, 407–412. 10.1016/j.clay.2013.07.006

[B31] Liu H. Yuan P. Qin Z. Liu D. Tan D. Zhu J. (2013). Thermal Degradation of Organic Matter in the Interlayer clay-organic Complex: A TG-FTIR Study on a Montmorillonite/12-Aminolauric Acid System. Appl. clay Sci. 80-81, 398–406. 10.1016/j.clay.2013.07.005

[B32] Niwa M. Katada N. Okumura K. (2010). Characterization and Design of Zeolite Catalysts: Solid Acidity, Shape Selectivity and Loading properties. Berlin, Germany: Springer Science & Business Media. 10.1007/978-3-642-12620-8

[B33] Novikova L. Belchinskaya L. Roessner F. (2013). Characterization of Surface Acidity and Catalytic Ability of Natural clay Minerals by Means of Test Catalytic Reaction. Acta Geodynamica et Geomaterialia 10 (4), 172. 10.13168/AGG.2013.0048

[B34] Ouellet-Plamondon C. Aranda P. Favier A. Habert G. van Damme H. Ruiz-Hitzky E. (2015). The Maya Blue Nanostructured Material Concept Applied to Colouring Geopolymers. RSC Adv. 5 (120), 98834–98841. 10.1039/c5ra14076e

[B35] Ovarlez S. Giulieri F. Delamare F. Sbirrazzuoli N. Chaze A.-M. (2011). Indigo-sepiolite Nanohybrids: Temperature-dependent Synthesis of Two Complexes and Comparison with Indigo-Palygorskite Systems. Microporous Mesoporous Mater. 142 (1), 371–380. 10.1016/j.micromeso.2010.12.025

[B36] Phukan A. Borah S. J. Bordoloi P. Sharma K. Borah B. J. Sarmah P. P. (2017). An Efficient and Robust Heterogeneous Mesoporous Montmorillonite clay Catalyst for the Biginelli Type Reactions. Adv. Powder Techn. 28 (6), 1585–1592. 10.1016/j.apt.2017.03.030

[B37] Polisi M. Arletti R. Morandi S. Fabbiani M. Martra G. Quartieri S. (2018). Zeolite/dye Hybrid Composites: Organization of Photoactive Azobenzene Molecules inside AlPO4-5. Microporous Mesoporous Mater. 268, 25–30. 10.1016/j.micromeso.2018.03.038

[B38] Pushpaletha P. Lalithambika M. (2011). Modified Attapulgite: An Efficient Solid Acid Catalyst for Acetylation of Alcohols Using Acetic Acid. Appl. Clay Sci. 51 (4), 424–430. 10.1016/j.clay.2010.12.033

[B39] Ramírez A. Sifuentes C. Manciu F. S. Komarneni S. Pannell K. H. Chianelli R. R. (2011). The Effect of Si/Al Ratio and Moisture on an Organic/inorganic Hybrid Material: thioindigo/Montmorillonite. Appl. clay Sci. 51 (1-2), 61–67. 10.1016/j.clay.2010.11.002

[B40] Rondão R. Seixas de Melo J. S. Bonifácio V. D. B. Melo M. J. (2010). Dehydroindigo, the Forgotten Indigo and its Contribution to the Color of Maya Blue. J. Phys. Chem. A. 114 (4), 1699–1708. 10.1021/jp907718k 20055403

[B41] Sánchez Del Río M. Martinetto P. Reyes-Valerio C. Dooryhée E. Suárez M. (2006). Synthesis and Acid Resistance of Maya Blue Pigment*. Archaeometry 48 (1), 115–130. 10.1111/j.1475-4754.2006.00246.x

[B42] Spange S. Prause S. Vilsmeier E. Thiel W. R. (2005). Probing Surface Basicity of Solid Acids with an Aminobenzodifurandione Dye as the Solvatochromic Probe. J. Phys. Chem. B 109 (15), 7280–7289. 10.1021/jp040521z 16851833

[B43] Tazi S. Rotenberg B. Salanne M. Sprik M. Sulpizi M. (2012). Absolute Acidity of clay Edge Sites from Ab-Initio Simulations. Geochimica et Cosmochimica Acta 94, 1–11. 10.1016/j.gca.2012.07.010

[B44] Van Olphen H. (1966). Maya Blue: A Clay-Organic Pigment? Science 154 (3749), 645–646. 10.1126/science.154.3749.645 17778806

[B45] Vandenabeele P. Bodé S. Alonso A. Moens L. (2005). Raman Spectroscopic Analysis of the Maya wall Paintings in Ek'Balam, Mexico. Spectrochimica Acta A: Mol. Biomol. Spectrosc. 61 (10), 2349–2356. 10.1016/j.saa.2005.02.034 16029856

[B46] Wang X. L. Zou G. W. Bi S. P. (2000). Advances in Determination of Aluminum in Environmental and Biological Materials by Al 27 Nuclear Magnetic Resonance Spectroscopy. Chin. J. Inorg. Chem. 16 (4), 548–560. 10.1103/PhysRevLett.60.658

[B47] Zheng S.-Q. Han Y. Huang X.-H. Dai Y.-L. Qian D. Zhang J.-C. (2010). Acid and Aluminium Modification of Sepiolite and its Application in FCC Catalysis. Clay miner. 45 (1), 15–22. 10.1180/claymin.2010.045.1.15

[B48] Zhuang G. Jaber M. Rodrigues F. Rigaud B. Walter P. Zhang Z. (2019). A New Durable Pigment with Hydrophobic Surface Based on Natural Nanotubes and Indigo: Interactions and Stability. J. Colloid Interf. Sci. 552, 204–217. 10.1016/j.jcis.2019.04.072 31129294

